# Evaluating national infection prevention and control minimum requirements: evidence from global cross-sectional surveys, 2017–22

**DOI:** 10.1016/S2214-109X(24)00277-8

**Published:** 2024-09-18

**Authors:** Ermira Tartari, Sara Tomczyk, Anthony Twyman, Ana Paula Coutinho Rehse, Mohamed Gomaa, Maha Talaat, Aparna Singh Shah, Howard Sobel, Joao Paulo Toledo, Benedetta Allegranzi

**Affiliations:** a Infection Prevention and Control Hub and Task Force, Department of Integrated Health Services, WHO, Geneva, Switzerland; b Faculty of Health Sciences, University of Malta, Msida, Malta; c Department for Infectious Disease Epidemiology, Robert Koch Institute, Berlin, Germany; d Infectious Hazard Management Programme, Health Emergencies Programme, WHO Regional Office for Europe, Copenhagen, Denmark; e WHO Regional Office for the Eastern Mediterranean, Cairo, Egypt; f Health Surveillance, Disease Prevention and Control, WHO Regional Office for South-East Asia, New Delhi, India; g Ministry of Health, WHO, Honiara, Solomon Islands; h High Impact Epidemics, WHO Health Emergencies Programme, WHO, Geneva, Switzerland

## Abstract

**Background:**

WHO infection prevention and control (IPC) minimum requirements provide standards to reduce the risk of infection during health-care delivery. We aimed to investigate the global implementation of these requirements at national levels and the progress of doing so across 2021–22 compared with 2017–18 to identify future directions for interventions.

**Methods:**

National IPC focal points were invited to complete an online survey measuring IPC minimum requirements from July 19, 2021, to Jan 31, 2022. The primary outcome was the proportion of countries meeting IPC minimum requirements. Country characteristics associated with this outcome were assessed with beta regression. Subset analyses were conducted to compare the 2021–22 indicators with a WHO IPC survey conducted in 2017–18 and to assess the correlation of the proportion of IPC minimum requirements met with the results of other WHO metrics.

**Findings:**

106 countries (ie, 13 low income, 27 lower-middle income, 33 upper-middle income, and 33 high income) participated in the survey (56% response rate). Four (4%) of 106 met all IPC minimum requirements. The highest scoring IPC core component was multimodal improvement strategies and the lowest was IPC education and training. The odds of meeting IPC minimum requirements was higher among high-income countries compared with low-income countries (adjusted odds ratio 2·7, 95% CI 1·3–5·8; p=0·020). Compared with the 2017–18 survey, there was a significant increase in the proportion of countries reporting an active national IPC programme (65% to 82%, p=0·037) and a dedicated budget (26% to 44%, p=0·037). Evaluation of the IPC minimum requirements compared with other survey instruments revealed a low positive correlation.

**Interpretation:**

To build resilient health systems capable of withstanding future health threats, urgently scaling up adherence to WHO IPC minimum requirements is essential.

**Funding:**

WHO.

**Translations:**

For the French and Spanish translations of the abstract see Supplementary Materials section.

## Introduction

Health-care-associated infections and antimicrobial resistance (AMR) are major challenges for global public health.^1^, ^2^ A study of point prevalence surveys in 99 countries worldwide between 2010 and 2020 reported that high-priority, antimicrobial-resistant pathogens cause 136 million health-care-associated infections annually.^3^ Notably, the European Centre for Disease Prevention and Control estimated that health-care-associated infections cause twice the amount of disability and premature mortality than 32 other infections combined.^2^

Effective infection prevention and control (IPC) programmes are crucial to reduce the burden of health-care-associated infections and AMR, and improve the quality of care and the safety of patients and health workers. IPC interventions can reduce health-care-associated infection rates by 35–70%.^4^, ^5^ A 2023 report of the Organisation for Economic Co-operation and Development found that scaling up IPC interventions in health-care settings can generate savings of up to US$11·7 billion purchasing power parity per year.^6^ The COVID-19 pandemic highlighted the importance of effective IPC programmes, with reports suggesting that up to 41% of inpatients were infected by SARS-CoV-2 during the first wave.^4^, ^7^, ^8^

WHO issued global recommendations on core components for IPC programmes at the national and health-care facility levels in 2016^9^ and on IPC minimum requirements to provide minimum protection and safety to patients, health workers, and visitors in 2019.^10^ Data from a WHO global survey in 2017–18 highlighted deficiencies in the implementation of national programmes, with 37·3% of countries lacking a functional IPC programme. Regional variations in IPC capacities were also observed, particularly according to the country income level.^11^

**Research in context**

**Evidence before this study**

We searched PubMed, MEDLINE, Web of Science, WHO's global health databases, and Google Scholar for peer-reviewed and preprint articles, published between Jan 1, 2000, and Feb 23, 2024, that reported international or multi-country assessments of infection prevention and control (IPC) programmes at the national level according to the WHO-recommended core components and the minimum requirements for IPC, without any language restrictions. We used the search terms “healthcare-associated infection”, “antimicrobial resistance”, “infection prevention and control” OR “infection prevention”, OR “infection control” OR “IPC”, “core components”, “minimum requirements”, “national programme” OR “national policy” OR “national progress”, and similar terms. We identified one international report issued by WHO that assessed the implementation of IPC core components at the national level across 88 countries worldwide during 2017–18. We found that 55 (63%) of 88 countries had a national IPC programme with an appointed IPC focal point, but only 11 (13%) reported the presence of all six key core component indicators and several gaps were identified. Other studies that assessed IPC programme implementation were uniquely focused on either the health-care facility level or exclusively assessed only a few specific aspects of IPC programmes. To the best of our knowledge, no comprehensive global study of IPC minimum requirements has been conducted to date.

**Added value of this study**

This study reports findings from the first WHO survey assessing the implementation of minimum requirements for IPC at the national level from all six WHO regions (Africa, the Americas, Eastern Mediterranean, Europe, South-East Asia, and the Western Pacific) and The World Bank income levels. It also includes a comparative analysis of the indicators derived from the 2021–22 survey with those of a previous survey and explores the correlation with concurrent IPC survey instruments. Our findings add value in various ways. First, as a cross-sectional assessment to monitor future progress at the global, regional, and national levels. Second, they can serve to steer the further development and implementation of IPC programmes or to identify areas for improvement within IPC programmes and to act as an accountability mechanism, driving national commitments and political action where this is still lacking.

**Implications of all the available evidence**

Our results showed substantial variability in the implementation of IPC minimum requirements among 106 countries worldwide and highlighted the need to improve relevant national governance responses. We also identified specific minimum requirements for each IPC core component that called for improved implementation. These data suggest that an intensified international response is needed to sustain some of the IPC progress achieved during the COVID-19 pandemic and to address the scale and severity of health-care-associated infections and antimicrobial resistance burden worldwide and the threat from emerging and re-emerging infectious pathogens, including efforts to monitor and evaluate IPC indicators and the education and training of health and care workers. This is of particular concern in low-income and middle-income countries where activities often lack dedicated financing for IPC activities.

A seminal milestone was reached during the 75th World Health Assembly in 2022, with a resolution agreed upon to improve IPC at national, subnational, and health-care facility levels. This was followed by the development of a global strategy and a global action plan and monitoring framework for IPC.^12^ Leading up to this resolution, we aimed to carry out a global survey to assess the implementation of minimum requirements for IPC programmes at national levels. We also aimed to evaluate temporal changes of IPC indicators across the WHO 2017–18 and 2021–22 global surveys^11^ as well as to examine the results of these surveys compared with other WHO tracking methods assessing IPC programme implementation.

## Methods

### IPC assessment tool for minimum requirements survey instrument

The IPC assessment tool for minimum requirements is a self-administered questionnaire adapted from the WHO national IPC assessment tool 2, which is designed to evaluate the minimum requirements for IPC programme implementation at the national level.^10^ The IPC assessment tool for minimum requirements comprises 25 dichotomous (ie, yes or no) indicators across six sections, mirroring the WHO IPC core components: (1) IPC programme, (2) IPC guidelines, (3) IPC education and training, (4) health-care-associated infection surveillance, (5) multimodal strategies, and (6) monitoring and audit of IPC practices, and feedback. These indicators were established through expert consensus and evidence, grounded in WHO's guidelines on core components for IPC^9^ as the foundational framework. The IPC assessment tool for minimum requirements uses a binary scoring method, where in the total score is the sum of yes responses to ensure the implementation of all IPC elements, without numerical cutoffs. The tool, including its scoring method, has been validated through external evaluation and pilot testing.

### Study design and participants

From July 19, 2021, to Jan 31, 2022, WHO conducted a global cross-sectional survey using the IPC assessment tool for minimum requirements survey instrument available in Arabic, Chinese, English, French, Russian, and Spanish. Data were collected using an online platform accessible through the WHO global IPC portal. The platform was pilot-tested in three low-income and high-income countries in December, 2020. National focal points for IPC in ministries of health or other governmental organisations of the 194 WHO member states were invited to participate by email and submit a single consolidated response per country. Study participation was voluntary and a targeted sampling approach was used to first include the 88 countries that participated in the 2017–18 WHO global national survey^11^ to enable a comparative analysis between the two survey periods. Furthermore, to aim for global representativeness, the target population of countries (n=194) was proportionally stratified by WHO region and The World Bank country income levels,^13^ with a target of reflecting 50% of the total breakdown within each regional income group.

The study was approved by the WHO Ethics Review Committee (ERC 0003629). Because it was a national assessment and did not include individual patient or health worker data, consent was not applicable. Instructions included information on data use and confidentiality (appendix 3 pp 2–5). Confidentiality was ensured and access was restricted to the research team at the WHO IPC Hub and the Task Force at Geneva headquarters (Switzerland).

### Comparison with other IPC survey instruments and previous data

To examine the relationship between our results on national IPC programme implementation and the findings of other monitoring systems, including facility-level data, we compared the 2021–22 global survey with other WHO methods, used as proxies for assessing IPC programme implementation (appendix 3 p 6): (1) 2017–18 WHO IPC survey at national level,^11^ (2) 2019 WHO IPC assessment framework survey at health-care facility level,^14^, ^15^ (3) 2021 International Health Regulations mandatory Electronic State Party Self-Assessment Annual Reporting IPC indicator,^16^ (4) 2021 Tripartite AMR Country Self-Assessment Survey indicator 8.1 (IPC in human health care),^17^ and (5) 2021 WHO and UNICEF's Joint Monitoring Programme data on water, sanitation, and hygiene (WASH) and environmental cleaning indicators at a national level.^18^

### Outcomes

The primary outcome was the proportion of countries fulfilling WHO IPC minimum requirements at a national level. The minimum requirements are defined as IPC standards that should be in place to provide minimum protection and safety to patients, health-care workers, and visitors, based on the WHO core components for IPC programmes.^10^ Secondary outcomes included the comparison of related indicators from the present 2021–22 survey to the other previously mentioned WHO methods assessing IPC programme implementation (see the defined proxies in the previous paragraph).

### Statistical analysis

The characteristics of survey respondents are described by WHO region and The World Bank country income levels.^13^ Absolute frequencies and proportions of countries meeting WHO specified minimum requirements for national IPC programmes were reported overall and individually, including by IPC core component, region, and income level. Differences by income level were assessed using Fisher's exact test. Using the Wilson score interval method, 95% CIs were generated for proportions. To assess the association between country characteristics and the proportion of countries meeting IPC programme minimum requirements, beta regression models were used to model the proportional binary response data, including an assessment of multicollinearity and model performance (appendix 3 p 7). Evaluated country characteristics included region, income level, and domestic general government health expenditure as a percentage of the gross domestic product from the WHO Global Health Expenditure Database,^19^ and the number of doctors and nurses per 10 000 population from the WHO Global Health Observatory.^20^ Changes in selected indicators from WHO national IPC surveys in 2017–18 and 2021–22 were assessed using McNemar's test for paired data. Subset analyses were conducted to assess the correlation of the proportion of reported IPC programme minimum requirements to the target indicators of other WHO and UN IPC survey instruments using the Pearson correlation coefficient (*R*^2^) with 95% CIs. Missing data were addressed by restricting the respective analyses to fully completed surveys. All analyses were done using the R statistical programme (version 4.3.2).

### Role of the funding source

WHO conceived the study design and carried out data collection, analysis, interpretation, and writing of the manuscript.

## Results

**Figure 1:**
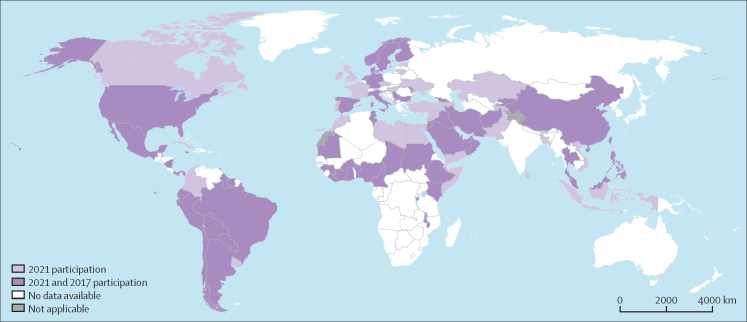
Country origin of survey responses included in the analysis of the global survey on minimum requirements for infection prevention and control, 2021 The total number of countries was 106; the number of countries participating in the 2017–18 global survey was 88.

From July 2021, to January, 2022, responses were received from 106 (55%) of 194 WHO member states (figure 1). Country stratification by WHO region was as follows: Africa 38% (18 of 47), the Americas 57% (20 of 35), Europe 64% (34 of 53), Eastern Mediterranean 100% (22 of 22), South-East Asia 55% (6 of 11), and the Western Pacific 22% (6 of 27). A greater proportion of countries from high-income (33 [53%] of 62) and upper-middle-income (33 [61%] of 54) levels participated than those in lower-middle-income (27 [55%] of 49) and low-income (13 [45%] of 29) levels.

Only four (4%) of 106 countries met 100% of IPC programme minimum requirements, 48 (45%) met 75% of the requirements, and 81 (76%) met a minimum threshold of 50% of the minimum requirements. No lower-middle-income or low-income country met 100% of the minimum requirements, whereas 3% (1 of 33) of upper-middle-income and 9% (3 of 33) of high-income countries met 100%. The European (17 [50%] of 34) and Western Pacific (3 [50%] of 6) regions had the highest proportions of countries meeting 75% minimum requirements, although more than 80% of countries in the Americas (17 of 20), the Western Pacific (five of six), and the African (15 of 18) regions met more than 50% of requirements. When adjusted only for income and region, high-income countries had higher odds of meeting a greater proportion of IPC minimum requirements than low-income countries (adjusted odds ratio 2·7, 95% CI 1·3–5·8). The WHO geographical region was not a significant predictor (appendix 3 p 7). When domestic general government health expenditure and the number of medical doctors, nursing, and midwifery personnel per 10 000 population were added to the model, no significant predictors were found (appendix 3 p 7).

**Figure 2:**
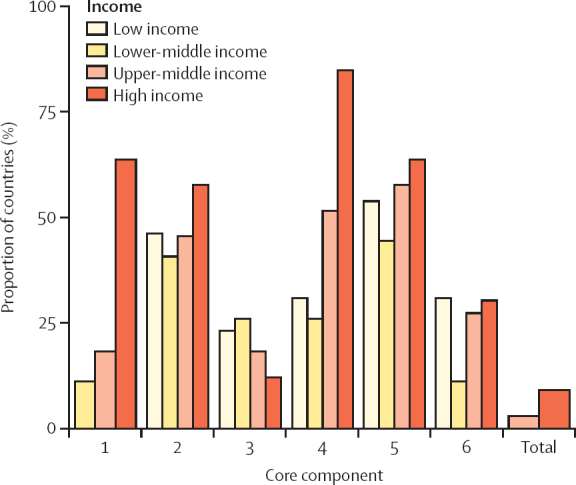
Proportion of countries meeting all reported minimum requirements by core component and World Bank country income level (N=106) No low-income countries met all indicators for core component 1 and no low-income and lower-middle-income countries met all indicators overall for the total; thus, no bars are shown for these latter groups.

The highest proportion of countries meeting all minimum requirements by core component was as follows: core component 5 (multimodal strategies; 59 [56%; 95% CI 45·7–65·2] of 106); core component 4 (health-care-associated infection surveillance; 56 [53%, 42·9–62·5]); and core component 2 (IPC guidelines; 51 [48%, 38·4–58·0]). These were followed by core component 6 (monitoring, audit, and feedback) and core component 1 (IPC programmes) with 26 (25%, 95% CI 16·9–34·0) of 106 countries and 30 (28%, 20·2–38·0) countries, respectively, with core component 3 (IPC education and training) scoring the lowest, with only 20 (19%, 12·2–27·9) of 106 countries meeting all minimum requirements (figure 2; table 1; see appendix 3 p 8 for further details on the correlation of core components met).

**Table 1:** Proportion of countries with reported established IPC minimum requirements by World Bank income level

	**All countries (n=106)**	**Comparison between The World Bank income levels**				p value*
		High-income countries (n=33)	Upper-middle-income countries (n=33)	Lower-middle-income countries (n=27)	Low-income countries (n=13)	
**Core component 1—IPC programme**						
An active IPC programme exists at the national level*	83 (78%)	28 (85%)	25 (76%)	19 (70%)	11 (85%)	0·56
An appointed IPC focal point in charge of the programme can be identified	97 (92%)	31 (94%)	29 (88%)	25 (93%)	12 (92%)	0·89
Focal points are trained in IPC and HAI prevention	85 (80%)	30 (91%)	25 (76%)	18 (67%)	12 (92%)	0·073
A protected and dedicated budget is allocated for IPC	43 (41%)	23 (70%)	10 (30%)	8 (30%)	2 (15%)	<0·0001
IPC focal points—at least one full-time equivalent	67 (63%)	24 (73%)	20 (61%)	16 (60%)	7 (54%)	0·56
**Core component 2—guidelines**						
The national IPC programme has a mandate to produce guidelines	96 (91%)	29 (88%)	29 (88%)	26 (96%)	12 (92%)	0·66
Use of evidence-based knowledge and internationally recognised national standards	90 (85%)	31 (94%)	28 (85%)	22 (81%)	9 (69%)	0·17
Guidelines for national coverage (all acute health-care facilities, public, and private)	94 (89%)	30 (91%)	29 (88%)	24 (89%)	11 (85%)	0·97
Guidelines reviewed and updated every 5 years	69 (65%)	25 (76%)	20 (61%)	17 (63%)	7 (54%)	0·44
Guideline adaptation and standardisation reflects local conditions	77 (73%)	27 (82%)	22 (67%)	18 (67%)	10 (77%)	0·47
**Core component 3—education and training**						
Guidance and recommendations provided for in-service IPC training	76 (72%)	23 (70%)	23 (70%)	20 (74%)	10 (77%)	0·95
Support for IPC training of health workers at the facility level	87 (82%)	26 (79%)	26 (79%)	24 (89%)	11 (85%)	0·77
A national IPC curriculum for in-service training of health-care workers has been developed	42 (40%)	13 (39%)	9 (27%)	14 (52%)	6 (46%)	0·25
A national system on the effectiveness of training and education is in place	30 (28%)	6 (18%)	10 (30%)	10 (37%)	4 (31%)	0·42
**Core component 4—surveillance**						
A multidisciplinary technical group for HAI surveillance is established	69 (65%)	30 (91%)	23 (70%)	10 (37%)	6 (46%)	<0·0001
A national strategic plan for HAI surveillance is in place	66 (62%)	28 (85%)	22 (67%)	10 (37%)	6 (46%)	<0·0001
IPC focal point team is trained in HAI surveillance	81 (76%)	32 (97%)	27 (82%)	13 (48%)	9 (69%)	<0·0001
**Core component 5—multimodal strategies**						
A trained national IPC focal point that is knowledgeable in implementation science and multimodal improvement strategies	77 (73%)	27 (82%)	22 (67%)	19 (70%)	9 (69%)	0·52
Coordinate and support local implementation of IPC improvement	79 (75%)	24 (73%)	27 (82%)	18 (67%)	10 (77%)	0·59
Multimodal strategies are promoted	75 (71%)	26 (79%)	21 (64%)	19 (70%)	9 (69%)	0·62
**Core component 6—monitoring, evaluation, and feedback**						
Established multidisciplinary technical group for IPC monitoring is in place	66 (62%)	27 (82%)	20 (61%)	12 (44%)	7 (54%)	0·020
A strategic plan for IPC monitoring is in place	55 (52%)	24 (73%)	16 (48%)	9 (33%)	6 (46%)	0·020
A minimal set of core indicators for health-care facilities in the country is defined	83 (78%)	29 (88%)	27 (82%)	18 (67%)	9 (69%)	0·19
A mechanism to train national and local auditors is in place	49 (46%)	19 (58%)	14 (42%)	9 (33%)	7 (54%)	0·26
Hand hygiene compliance monitoring and feedback is a key national indicator	70 (66%)	23 (70%)	22 (67%)	16 (59%)	9 (69%)	0·86

Data are n (%), unless otherwise specified. A full list of IPC minimum requirement indicators is provided in appendix 3 (pp 9–10). IPC=infection prevention and control. HAI=health-care-associated infection.

* Active is defined as a functioning programme with annual work plans and a budget.

An existing active and functioning national IPC programme with annual work plans and a budget was reported in 83 (78%, 95% CI 69·0–85·5) of 106 countries (table 1). Only 43 (41%, 31·3–50·6) countries reported a dedicated budget allocated to the IPC programme with significant income level differences observed (low income 2 [15%] of 13 *vs* high income 23 [70%] of 33]; p<0·0001; table 1).

For training and education, 42 (40%, 95% CI 30·4–49·6) of 106 countries reported having a national IPC curriculum for in-service training developed in alignment with national guidelines and approved by national bodies (table 1). 30 countries (28%, 20·2–38·0) across all income levels reported having a national system for monitoring the effectiveness of IPC training and education at least annually. 75 (71%, 61·0–79·0) of countries reported that multimodal improvement strategies were included in national IPC guidelines and education and training, with no differences across income levels (table 1). 66 countries (62%, 52·3–71·3) reported having a national strategic plan for health-care-associated infection surveillance developed by a multidisciplinary technical group (table 1).

66 countries (62%, 52·3–71·3) reported having a national-level multidisciplinary technical group for IPC monitoring. Significant disparities were consistently observed in these indicators across income levels, in particular in low-income versus high-income countries (table 1).

62 countries participated in both WHO 2017–18 and 2021–22 national global surveys. Most related IPC indicators significantly improved between the two survey periods (table 2), including the percentage of countries reporting an active national IPC programme (40 [65%] to 51 [82%], p=0·037), a dedicated and protected national IPC budget (16 [26%] to 27 [44%], p=0·037), and IPC guidelines developed from international standards (42 [68%] to 57 [92%], p<0·0007). Countries also reported a significant increase in indicators related to the presence of dedicated and trained IPC focal points (13 [21%] to 40 [65%], p<0·0001), the promotion of multimodal improvement strategies for implementing IPC practices (33 [53%] to 49 [79%], p=0·0046), and monitoring of hand hygiene compliance (19 [31%] to 45 [73%], p<0·0001). By contrast, the proportion of countries reporting indicators related to the overall monitoring of IPC-related indicators did not significantly change (41 [66%] to 37 [60%], p=0·69). Indicators related to a national in-service IPC education curriculum decreased from 58% (n=36) in 2017–18 to only 40% (n=25) in 2021 (p=0·045; table 2).

**Table 2:** Selected comparison of first and second national IPC global surveys by indicator (N=62)

	**First national survey indicator (2017)**	**n (%)**	**Second national survey indicator (2021)**	**n (%)**	p value*
**IPC domain**					
IPC programme	There is a national IPC programme	40 (65%)	An active IPC programme exists at the national level	51 (82%)	0·037
Budget	The IPC team has a protected and dedicated budget	16 (26%)	There is an identified, protected, and dedicated budget allocated to the IPC programme according to planned activity	27 (44%)	0·037
International standards	Guidelines are developed from international standards	42 (68%)	The development of guidelines involves the use of evidence-based scientific knowledge and international national standards	57 (92%)	<0·0007
Curriculum	There is an in-service IPC curriculum	36 (58%)	A national IPC curriculum for in-service training of health-care workers has been developed in alignment with the national IPC guidelines approved and endorsed by an appropriate national body	25 (40%)	0·045
Monitoring	IPC-related indicators are monitored at a national level	41 (66%)	A strategic plan for IPC monitoring is in place, including an integrated system for the collection and analysis of data	37 (60%)	0·69
Hand hygiene	Hand hygiene compliance is monitored at a national level	19 (31%)	Hand hygiene compliance monitoring and feedback is identified as a key national indicator at the very least for reference hospitals	45 (73%)	<0·0001
**Other related indicators**					
IPC team	The IPC team includes one or more dedicated professionals (with no shared responsibilities to other departments)	13 (21%)	The appointed IPC focal points have undergone training in IPC in the prevention of HAI	40 (65%)	<0·0001
Guidelines	There are national IPC guidelines	45 (73%)	The national IPC programme has a mandate to produce guidelines for preventing and controlling HAI	57 (92%)	0·014
HAI surveillance	There is a national programme or system for HAI surveillance	28 (45%)	A national strategic plan for HAI surveillance (with a focus on priority infections based on the local context) has been developed by the multidisciplinary technical group	41 (66%)	0·012
Multimodal strategies	The national IPC team supports multimodal strategies to implement IPC practices at the facility level	33 (53%)	Multimodal strategies are promoted through the inclusion of the approach in the development of IPC guidelines, education, and training	49 (79%)	0·0046

Indicators were intended to measure the same constructs or domains in each survey, but with slight variations of wording. Of the included countries, 10 were low income, 14 were lower-middle income, 18 were upper-middle income, and 20 were high income. Included countries by WHO region were: African region (n=16)—Benin, Burkina Faso, Burundi, Cameroon, Chad, Côte D'Ivoire, Ethiopia, Ghana, Guinea, Kenya, Liberia, Malawi, Mauritania, Nigeria, Uganda, and Zimbabwe; Eastern Mediterranean region (n=12)—Afghanistan, Bahrain, Iran, Iraq, Jordan, Kuwait, Oman, Qatar, Saudi Arabia, Sudan, United Arab Emirates, and Tunisia; European region (n=14)—Bulgaria, Denmark, Finland, Georgia, Germany, Italy, Kyrgyzstan, Malta, Moldova, the Netherlands, Norway, Serbia, Spain, and Sweden; Region of the Americas (n=15)—Argentina, Bolivia, Brazil, Chile, Ecuador, Guyana, Jamaica, Mexico, Nicaragua, Panama, Paraguay, Peru, Suriname, Trinidad and Tobago, and the USA; Western Pacific region (n=4)—China, Malaysia, the Philippines, and Singapore; and South-East Asia region (n=1)—Thailand. IPC=infection prevention and control. HAI=health-care-associated infections.

* McNemar's test for paired data (with Bonferroni correction for multiple comparisons).

**Figure 3:**
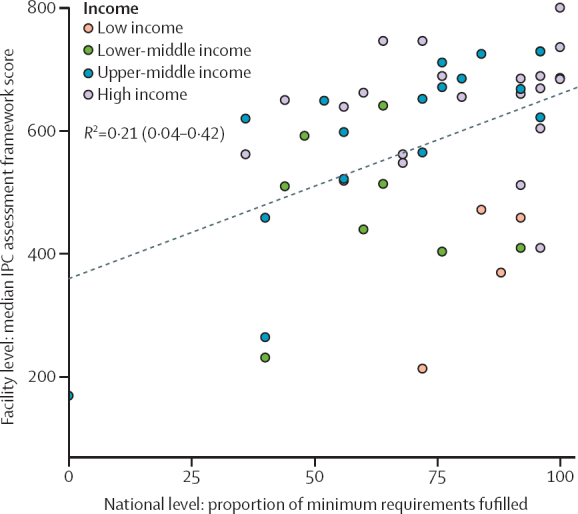
Correlation of weighted IPC assessment framework facility median scores at country level and the proportion of IPC minimum requirements met at the national level among countries participating in both surveys (N=51) The included countries are those among the 106 participating countries in the national survey and 78 countries with health-care facilities who participated in the IPC assessment framework global survey and met the inclusion criteria (ie, they completed all core component indicator questions, the threshold for which was based on the number of survey responses per capita, and weighting based on The World Bank country income level, WHO region, facility care level [primary, secondary, or tertiary], and type of facility [private or public]). IPC=infection prevention and control.

51 countries participated in both the 2019 global IPC assessment framework survey at the acute health-care facility level and the present 2021–22 global survey at a national level. When compared, there was a low degree of positive correlation observed between the weighted total IPC assessment framework score at the acute health-care facility level and the proportion of IPC minimum requirements met at the national level (*R*^2^=0·21, 95% CI 0·04–0·42; figure 3). A comparison of the present survey and the available e-SPAR International Health Regulations data^16^ (n=101) indicated weak correlations between the proportion of IPC minimum requirements met at national level and e-SPAR scores for the indicators C9 overall IPC (*R*^2^=0·15, 95% CI 0·04–0·29), C9.1 IPC programmes (0·17, 0·06–0·32), and C9.2 surveillance (0·10; 0·02–0·23).

When comparing responses to this survey and the Tripartite AMR Country Self-Assessment Survey AMR reporting system in 2021 (n=100 countries), the median proportion of IPC minimum indicators met in the present survey increased as the Tripartite AMR Country Self-Assessment Survey indicator for IPC programme capacity increased from level A (48%, IQR 40–72) to E (92%, 79–96; appendix 3 p 9). However, some conflicting country responses were also observed between the two surveys. Among countries who reported A: no national IPC programme or operational plan available in the Tripartite AMR Country Self-Assessment Survey, six (67%) of nine stated that an active IPC programme existed at the national level in the present survey. No statistically significant associations were found between WASH indicators and the proportion of IPC minimum requirements met.

## Discussion

This global survey provides a comprehensive snapshot of IPC national programme implementation in 106 countries across all six WHO regions and The World Bank income levels. To the best of our knowledge, this is the first study to assess the international implementation of IPC minimum requirements at the national level and evaluate implementation changes over time. The survey found an overall increase in the proportion of countries implementing a national IPC programme from 2017–18 to 2021–22, as well as improvements in several IPC indicators. Despite these positive trends and growing recognition of IPC as a fundamental aspect of global health security, major gaps in national efforts remain across different income levels. By providing a comprehensive and systematic examination of the status of IPC programme core components, this study highlights priority areas for improvement to help countries prepare for future public health emergencies.

83 (78%) of 106 countries reported having established an active national IPC programme, but only four (4%) met all minimum requirements of IPC core components. This stark contrast highlights a substantial gap between the initiation of IPC programmes and the further implementation of all minimum requirements to make IPC programmes effective. There were also significant variations in implementation according to country income levels, with no low-income or lower-middle-income countries meeting all minimum requirements. Notably, our findings align with previous WHO global survey results at the facility level, which reported only 15% of 4440 health-care facilities in 81 countries meeting all minimum requirements, of which none were low-income countries.^15^ The recurrent observation that high-income countries exhibit a higher likelihood of meeting IPC standards underscores an urgent need to reinforce the crucial link between financial resources and IPC implementation success, particularly in countries with limited resources. It also prompts a reevaluation of how support is structured and delivered to meet local needs and disparities across health-care systems.

Contrary to our findings, which identified the use of multimodal strategies for implementing IPC practices as the core component with the highest proportion of countries reporting meeting all minimum requirements, previous studies have documented challenges in understanding and implementing these strategies, including in high-income settings.^11^, ^21^ Furthermore, a comparison of the 2017–18 and 2021–22 surveys showed a notable improvement in promoting multimodal strategies.^15^ This trend is encouraging as evidence indicates that these strategies are the most effective means of implementing IPC interventions within IPC programmes.^4^, ^9^, ^22^, ^23^ This could be partly due to WHO's concerted efforts in promoting multimodal improvement strategies for IPC and supporting country capacity building following the release of the IPC core component guidelines, which could have influenced the favourable trend observed in our analysis.^24^

Our study, as well as previous evidence and the COVID-19 pandemic,^25^ highlight the urgent need to improve education and training in IPC for both health-care workers’ and patient protection.^11^, ^15^ Although scattered IPC education initiatives exist, a more sustained and structured approach is required. The absence of a national IPC curriculum for the ongoing training of all health-care workers and the scarcity of established mechanisms for regularly assessing training effectiveness is often observed.^15^ Implementing effective monitoring and reporting of health-care-associated infections and IPC-related indicators is integral to evaluating prevention and control measures within health systems and crucial to implementing necessary changes. However, a lack of trained personnel and gaps in access to quality-controlled diagnostics and reliable data systems pose barriers to surveillance, particularly in low-resource settings.^21^, ^26^, ^27^ Significant discrepancies in health-care-associated infection surveillance implementation across income levels emphasise the need for targeted capacity-building efforts in epidemiology and microbiology and improved access to related diagnostics and data tools, especially in low-resource settings. Reports indicate that core component 4 (health-care-associated infection surveillance) and core component 6 (monitoring, audit of IPC practices, and feedback) have the lowest scores among low-income countries.^3^, ^11^, ^15^, ^27^

There was a significant increase from 2017–18 to 2021–22 in the proportion of countries reporting the implementation of an active IPC programme and an appointed, trained, IPC focal point with dedicated time to support the programme at a national level, suggesting that the COVID-19 pandemic might have accelerated the pace of global IPC programme implementation.^5^ However, evidence is relatively scarce with some studies reporting increased IPC capacity in response to the pandemic.^28^, ^29^, ^30^

While some countries have swiftly designated IPC focal points without establishing a national programme, the definitions of an active IPC programme and focal point might vary across countries. The emphasis should be on what comprises an effective programme, as evidence has shown that active national programmes can substantially reduce health-care-associated infections.^4^, ^9^

Given the impetus generated by the COVID-19 pandemic, there is evidence of a heightened interest and tangible progress in the implementation of the minimum requirements by WHO and core components of IPC programmes, actively endorsed by other crucial stakeholders. The allocation of sufficient funding for IPC programmes is of the utmost importance, including human resources and infrastructure. A comparison of budget allocations for IPC activities between 2017–18 and 2021–22 showed an increase, likely due to the direct response to the COVID-19 pandemic. While this surge in funding could provide immediate benefits, long-term sustainability of these investments is a cause for concern.^29^ Lessons learned from COVID-19 and other outbreaks highlight the need to maintain momentum and continued support for IPC programmes to build an adequate infrastructure, including education and training for health workers and a capacity for health-care-associated infection surveillance and IPC monitoring.^5^, ^15^ Low-income and lower-middle-income countries could require support from foreign donors or private philanthropy to acquire sufficient financing, necessitating renewed international collaboration and public–private partnerships.

Our findings suggest a low positive correlation between the IPC score at the acute health-care facility level and the extent to which minimum requirements are met at the national level. The granularity and specificity of what each survey measures can vary substantially, which might lead to discordant results. Further, discrepancies in the timing of data collection or changes in the IPC landscape over time could affect correlation. Indeed, the IPC facility-level survey was conducted in 2019, almost two years before the conduct of the national assessment and before the COVID-19 pandemic. The low correlation between the proportion of IPC minimum requirements met at the national level and the e-SPAR scores for indicators related to IPC programmes and safe environments in health-care facilities might also have been due to the fact that the surveys were not conducted exactly at the same time (although in the same year). Furthermore, differences in interpretation of the indicators could have occurred as the surveys were likely completed by different people; for example, the national International Health Regulations focal point completing the e-SPAR is a different person from the IPC national focal point in most, if not all, countries. Finally, no significant associations were found with WASH factors, which can be partly explained by the fact that the national IPC survey instrument does not include WASH. A better standardisation of IPC indicators and methods used for self-assessment is necessary across existing assessment tools and systems, including training approaches.

Our study has some limitations. First, although the results deliver a valuable global perspective on the implementation of IPC minimum requirements, they might oversimplify complex experiences across countries and health-care systems. The response rate was relatively low, in particular from certain regions (Western Pacific), which could have reduced global representativeness. Second, the study relied on self-reported data from IPC national focal points, which could have been shaped by individual perceptions and access to national data. If national focal points did not coordinate their responses with different IPC-related stakeholders or lacked knowledge of country data, the validity and reliability of responses could have been compromised. Surveys are susceptible to social desirability bias in which respondents prefer to select the best answer over the true answer. Nonetheless, the confidential nature of the survey data collection and anonymous reporting might have mitigated the risk. Third, validation of country responses by regional IPC focal points was conducted only for the Eastern Mediterranean region and could have introduced a region-specific bias.

These study findings have substantial implications for global health policy and practice. A stronger governmental commitment to tackling health-care-associated infections and the effective implementation of IPC programmes is essential. Countries without established IPC programmes should prioritise their development to reduce the burden of health-care-associated infections and AMR. Crucially, these programmes should be given heightened and sustained prominence on political and policy making agendas beyond crisis periods, with an associated increased investment in resources, infrastructure, and training.

### Contributors

### Data sharing

The research protocol for this study (ERC 0003629) included a commitment by WHO to restrain from publicly sharing results per country to improve participation and minimise social desirability bias. Since aggregated results are by WHO region, The World Bank income levels are already available in the tables in the Article and in appendix 3; no other data will be shared.

## Declaration of interests

We declare no competing interests.

## Supplementary Materials

French translation of the abstract

Spanish translation of the abstract
