# Supplementary material for: Evaluating national infection prevention and control minimum requirements: evidence from global cross-sectional surveys, 2017–22
Source: Lancet Glob Health. 2024 Sep 18;12(10):e1620–8. doi: 10.1016/S2214-109X(24)00277-8 (PMC11420467; doi:10.1016/S2214-109X(24)00277-8)
Supplement: French translation of the abstract [file mmc1.pdf]

THE LANCET
Global Health

Supplementary appendix 1

This translation in French was submitted by the authors and we reproduce it as supplied. It has not been peer reviewed. *The Lancet*'s editorial processes have only been applied to the original in English, which should serve as reference for this manuscript.

Cette traduction en français a été proposée par les auteurs et nous l'avons reproduite telle quelle. Elle n'a pas été examinée par des pairs. Les processus éditoriaux du *Lancet* n'ont été appliqués qu'à l'original en anglais et c'est cette version qui doit servir de référence pour ce manuscrit.

Supplement to: Tartari E, Tomczyk S, Twyman A, et al. Evaluating national infection prevention and control minimum requirements: evidence from global cross-sectional surveys, 2017–22. *Lancet Glob Health* 2024; **12:** e1620–28.

Titre: Évaluation des exigences minimales nationales en matière de prévention et de contrôle des infections : preuves tirées d'enquêtes transversales mondiales, 2017–22.

Résumé

Contexte : Les exigences minimales de l'OMS en matière de prévention et de contrôle des infections (PCI) fournissent des normes pour réduire le risque d'infection lors des soins de santé. Nous avons cherché à étudier la mise en œuvre mondiale de ces exigences au niveau national et les progrès réalisés à cet égard entre 2021–22 par rapport à 2017–18, afin d'identifier les orientations futures pour les interventions.

Méthodes : Les points focaux nationaux de la PCI ont été invités à remplir un sondage en ligne mesurant les exigences minimales de la PCI de Juillet 19, 2021 à Janvier 31, 2022. Le principal résultat était la proportion de pays répondant aux exigences minimales de la PCI. Les caractéristiques des pays associées à ce résultat ont été évaluées par régression bêta. Des analyses de sous-ensembles ont été réalisées pour comparer les indicateurs de 2021–22 avec ceux d'une enquête de l'OMS sur la PCI réalisée en 2017–18 et pour évaluer la corrélation entre la proportion d'exigences minimales de la PCI respectées et les résultats d'autres indicateurs de l'OMS.

Résultats : 106 pays (soit 13 à faible revenu, 27 à revenu intermédiaire inférieur, 33 à revenu intermédiaire supérieur et 33 à revenu élevé) ont participé à l'enquête (taux de réponse de 56 %). Quatre (4 %) des 106 pays ont satisfait à toutes les exigences minimales de la PCI. Le composant de base de la PCI avec le meilleur score était les stratégies d'amélioration multimodales et le plus faible était la formation et l'éducation en matière de PCI. Les chances de respecter les exigences minimales de la PCI étaient plus élevées parmi les pays à revenu élevé par rapport aux pays à faible revenu (odds ratio ajusté 2,7, IC à 95 % 1,3–5,8 ($p = 0{,}009$)). Par rapport à l'enquête de 2017–18, il y a eu une augmentation significative du nombre de pays rapportant un programme national actif de PCI et un budget dédié ($p < 0{,}0001$). L'évaluation des exigences minimales de la PCI par rapport à d'autres instruments d'enquête a révélé une faible corrélation positive.

Interprétation : Pour construire des systèmes de santé résilients capables de résister aux menaces sanitaires futures, il est essentiel d'accroître de toute urgence l'adhésion aux exigences minimales de la PCI de l'OMS.
