# Supplementary material for: Evaluating national infection prevention and control minimum requirements: evidence from global cross-sectional surveys, 2017–22
Source: Lancet Glob Health. 2024 Sep 18;12(10):e1620–8. doi: 10.1016/S2214-109X(24)00277-8 (PMC11420467; doi:10.1016/S2214-109X(24)00277-8)
Supplement: Spanish translation of the abstract [file mmc2.pdf]

THE LANCET
Global Health

## Supplementary appendix 2

This translation in Spanish was submitted by the authors and we reproduce it as supplied. It has not been peer reviewed. *The Lancet*'s editorial processes have only been applied to the original in English, which should serve as reference for this manuscript.

Los autores nos proporcionaron esta traducción al español y la reproducimos tal como nos fue entregada. No la hemos revisado. Los procesos editoriales de *The Lancet* se han aplicado únicamente al original en inglés, que debe servir de referencia para este manuscrito.

Supplement to: Tartari E, Tomczyk S, Twyman A, et al. Evaluating national infection prevention and control minimum requirements: evidence from global cross-sectional surveys, 2017–22. *Lancet Glob Health* 2024; **12:** e1620–28.

**Título**: Evaluación de los requisitos mínimos nacionales de prevención y control de infecciones: evidencia de encuestas transversales globales, 2017–22.


**Resumen**

**Antecedentes**: Los requisitos mínimos de la OMS para la prevención y control de infecciones (PCI) establecen estándares para reducir el riesgo de infección durante la atención sanitaria. Nuestro objetivo fue investigar la implementación global de estos requisitos a nivel nacional y el progreso en su cumplimiento durante 2021–22 en comparación con 2017–18 para identificar direcciones futuras para intervenciones.

**Métodos**: Se invitó a los puntos focales nacionales de PCI a completar una encuesta en línea que midiera los requisitos mínimos de PCI desde Julio 19, de 2021 hasta Enero 31, de 2022. El resultado principal fue la proporción de países que cumplieron con los requisitos mínimos de PCI. Las características de los países asociadas con este resultado se evaluaron con regresión beta. Se realizaron análisis de subconjuntos para comparar los indicadores de 2021–22 con una encuesta de PCI de la OMS realizada en 2017–18 y para evaluar la correlación entre la proporción de requisitos mínimos de PCI cumplidos y los resultados de otros indicadores de la OMS.

**Resultados**: 106 países (es decir, 13 de bajos ingresos, 27 de ingresos medianos bajos, 33 de ingresos medianos altos y 33 de altos ingresos) participaron en la encuesta (tasa de respuesta del 56 %). Cuatro (4 %) de 106 países cumplieron con todos los requisitos mínimos de PCI. El componente central de PCI con la puntuación más alta fue las estrategias de mejora multimodal y el más bajo fue la educación y capacitación en PCI. Las probabilidades de cumplir con los requisitos mínimos de PCI fueron mayores en los países de altos ingresos en comparación con los países de bajos ingresos (razón de probabilidades ajustada 2,7, IC del 95 % 1,3–5,8 (p=0,009)). En comparación con la encuesta de 2017–18, hubo un aumento significativo en el número de países que informaron sobre un programa nacional activo de PCI y un presupuesto dedicado (p<0,0001). La evaluación de los requisitos mínimos de PCI en comparación con otros instrumentos de encuesta reveló una baja correlación positiva.

**Interpretación**: Para construir sistemas de salud resilientes capaces de resistir futuras amenazas para la salud, es esencial aumentar urgentemente la adhesión a los requisitos mínimos de PCI de la OMS.
