# Supplementary material for: Evaluating national infection prevention and control minimum requirements: evidence from global cross-sectional surveys, 2017–22
Source: Lancet Glob Health. 2024 Sep 18;12(10):e1620–8. doi: 10.1016/S2214-109X(24)00277-8 (PMC11420467; doi:10.1016/S2214-109X(24)00277-8)
Supplement: Supplementary appendix 3 [file mmc3.pdf]

THE LANCET
Global Health

Supplementary appendix 3

This appendix formed part of the original submission and has been peer reviewed. We post it as supplied by the authors.

Supplement to: Tartari E, Tomczyk S, Twyman A, et al. Evaluating national infection prevention and control minimum requirements: evidence from global cross-sectional surveys, 2017–22. *Lancet Glob Health* 2024; **12:** e1620–28.

Supplemental Table of Contents

Page 1. Table of Content
Page 2: Delphi round 1 and 2: percentage of approved statements per domain
Page 3: Institutional safety climate for hand hygiene improvement: Research statements excluded after Round 2
Page 4-5: Participant comments in Round 1 and Round 2
Page 6-8: Implementation of the research agenda: research priorities with suggested possible research questions, needed stakeholders, fundimg sources and possible research designs

Supplemental Table 1. Delphi round 1 and 2: percentage of approved statements per domain*

Domain areas	After Round 1	After Round 2
1. Institutional safety climate – general priorities	83% (5 of 6)	100% (6 of 6)
2. Personal accountability for hand hygiene	80% (8 of 10)	90% (9 of 10)
3. Leadership	100% (7 of 7)	100% (7 of 7)
4. Patient participation and empowerment	67% (6 of 9)	89% (8 of 9)
5. Religion and traditions	25% (1 of 4)	25% (1 of 4)

*Overall approval across all domains was 75% (31 of 36).

Supplemental Table 2. Institutional safety climate for hand hygiene improvement: Research statements excluded after Round 2

Domain	Excluded
1. Institutional safety climate – general priorities	• No exclusions.
2. Personal accountability for hand hygiene	• 2.10 Influence of different institutional social networks on personal accountability for hand hygiene.
3. Leadership	• No exclusions.
4. Patient participation and empowerment	• 4.9 Underlying ethical considerations in the development of a patient participation/ empowerment strategy as part of an overall approach to hand hygiene improvement.
5. Religion and traditions	• 5.1 Influence of different religions and traditions on hand hygiene improvement strategies, including as barriers and facilitators.<br>• 5.2 Relationship between religious beliefs, practices and traditions and institutional safety climate that influences hand hygiene.<br>• 5.4 Influence of religion and traditions on the acceptance and use of alcohol-based hand hygiene products.

Supplemental Table 3A: Examples of participant comments in Round 1

Domain area	Priorities	Example of comments
Institutional safety climate – general priorities	1.4 Influence of a health care facility's safety and quality climate/culture on hand hygiene practices during outbreaks/emergencies/pandemics.	Not sure how important this is. The safety culture should be applicable under regular and crisis situations.
Personal accountability for hand hygiene	2.2 Relationship between training of individual health workers and personal accountability for hand hygiene improvement.	I think to measure this association will be difficult for a few reasons: 1) how is personal accountability going to be measured, and 2) the link between training and the behaviour will be difficult to correlate given some or many other potentially confounding variables that might influence the behaviour. Though this research areas is feasible, cost effective, and important, I am not sure that the resultant data will actual bring forth additional data to develop future interventions.
Leadership	3.2 Most effective governance structures for shaping/influencing an institutional safety climate that supports hand hygiene.	Some of this is very context- and country-specific, whereas other topics have more potential for impact.
Patient participation and empowerment	4.5 Role and impact of visitors and informal caregivers in hand hygiene improvement.	Particularly important in some settings where care is provided by family members (low-/middle-income countries), but also in certain settings for high-income countries (eg, paediatrics, care of the elderly)-
Religion and traditions	5.1Influence of different religions and traditions on hand hygiene improvement strategies, including as barriers and facilitators.	Could religions and traditions be incorporated into the earlier research priority looking into barriers?

Supplemental Table 3B: Example of participant comments in Round 2

Domain	Priorities	Example comments
Institutional safety climate – general priorities	1.5 Role of media (mainstream and social media) in shaping/influencing an institutional safety climate and hand hygiene improvement.	It may be difficult to match the impact/influence of media (by definition, focused on large scale) on an individual organisation, which in itself is a microcosm.
Personal accountability for hand hygiene	2.8 Factors that influence the development (eg, training, mentoring, attitudes, beliefs, values) of an effective hand hygiene champion.	I think this is particularly important to improve the effectiveness of implementation process. Understanding key determinants of optimal adoption may help organisations not to engage with interventions until or unless they have encouraged the key determinants identified.
Leadership	Not applicable	Not applicable
Patient participation and empowerment	4.5 Perceptions of service users and patients towards an institutional safety climate and its impact on hand hygiene standard.s	This is not well evidenced currently so worthy of consideration on that basis.
Religion and traditions	5.1 Influence of different religions and traditions on hand hygiene improvement strategies, including as barriers and facilitators	Well, this one is so close to the 70% cut-off. I continue agreeing with it.

Supplemental Table 4: Implementation of the research agenda: research priorities with suggested possible research questions, needed stakeholders, funding sources and possible research designs

Research Priorities Table				
**Research Priority**	**Research Questions**	**Needed Stakeholders**	**Funding Sources**	**Possible Research Designs**
**Influence of different cadres of the health workforce on the institutional safety climate**	How do different health worker groups influence institutional safety climate?	Hospital management, research networks	Government health departments, hospital administrations	Focus groups, observational studies
**Perspectives of different cadres of health workers towards an institutional safety climate**	What are the views of various health worker groups on institutional safety climate?	Hospital management, research networks	Research grants, hospital administrations	Qualitative, exploratory, observational studies
**Relationship between a health care facility's safety and quality climate/culture and hand hygiene**	How does the safety and quality climate in a healthcare facility relate to hand hygiene?	Healthcare workers, infection control teams	Health organizations, hospital administrations	Longitudinal studies, cross-sectional studies, case studies
**Influence of healthcare facility's safety and quality climate/culture on hand hygiene during outbreaks/emergencies/pandemics**	How does the safety climate impact hand hygiene in outbreak/emergencies?	Infection control teams, research networks	Emergency preparedness funds, health organizations	Case studies, observational studies, emergency response evaluations
**Role of media (mainstream and social) in shaping/influencing an institutional safety climate and hand hygiene improvement**	How does media (mainstream and social) influence institutional safety climate and hand hygiene?	Media /social media organizations, infection control teams, public health officials	Public health organizations	Content analysis, media impact studies
**Role of hand hygiene campaigns in influencing a sustained institutional safety climate**	How do hand hygiene campaigns affect institutional safety climate?	Infection control teams, campaign organizers	Government health departments, non-governmental organizations (NGOs)	Campaign evaluations, surveys, longitudinal studies
**Best methods for measuring personal accountability in hand hygiene**	What are the most effective ways to measure personal accountability for hand hygiene?	Hospital management, infection control teams	Research grants, hospital administrations	Survey development, validation studies, experimental studies
**Relationship between training and personal accountability for hand hygiene improvement**	How does training impact personal accountability for hand hygiene?	Training providers, infection control teams	Hospital administrations, government training programs	Training evaluations, pre- and post-training surveys, longitudinal studies
**Influence of enabling environment on personal accountability for hand hygiene**	How does the built environment affect personal accountability for hand hygiene?	Hospital management, infection control teams	Hospital administrations, government infrastructure funds	Environmental assessments, observational studies, intervention studies
**Relationship between monitoring/feedback methods and personal accountability for hand hygiene**	How do different monitoring and feedback methods affect personal accountability?	Hospital management, infection control teams	Hospital administrations, research grants	Comparative studies, intervention studies, surveys
**Impact of appraisal/reward systems on personal accountability for hand hygiene**	What is the impact of appraisal and reward systems on personal accountability for hand hygiene?	Hospital management, healthcare workers	Hospital administrations, government health departments	Experimental studies, survey-based evaluations, case studies
**Relationship between perceptions of hand hygiene and personal accountability**	How do health workers' perceptions of hand hygiene relate to personal accountability?	Healthcare workers, infection control teams	Research grants, hospital administrations	Surveys, interviews, focus groups

**Influence of hand hygiene champions/role models on personal accountability**	How do hand hygiene champions and role models influence personal accountability?	Quality teams, infection control teams, hand hygiene champions	NGOs, hospital administrations, health organizations	Case studies, observational studies, surveys
**Factors influencing the development of an effective hand hygiene champion**	What factors contribute to developing an effective hand hygiene champion?	Training providers, mentors, infection control teams	Research grants, hospital administrations	Qualitative studies, training evaluations, surveys
**Relationship between leadership valuing hand hygiene and personal accountability**	How does leadership valuing hand hygiene affect personal accountability among health workers?	Hospital leadership, quality teams	Hospital administrations, government health departments	Leadership training evaluations, longitudinal studies, surveys
**Effectiveness of leadership approaches to improve institutional safety climate**	How effective are leadership approaches in improving institutional safety climate?	Hospital leadership, quality teams	Government health departments, hospital administrations	Leadership evaluations, case studies, comparative studies
**Most effective governance structures for supporting hand hygiene**	What are the most effective governance structures for supporting hand hygiene?	Hospital leadership, policymakers	Government health departments, hospital administrations	Policy analysis, case studies, surveys
**Barriers and drivers to institutionalizing hand hygiene as a priority**	What are the barriers and drivers to making hand hygiene a priority at the institutional level?	Hospital management, infection control teams	Research grants, hospital administrations	Qualitative studies, barrier and facilitator analysis, surveys
**Influence of IPC/hand hygiene training on institutional safety climate**	How does IPC/hand hygiene training for hospital leaders influence institutional safety climate?	Hospital leadership, training providers, infection control teams	Government health departments, hospital administrations	Training evaluations, pre- and post-training surveys, case studies
**Direct relationship between leadership support for hand hygiene and improvement/performance**	How does leadership support for hand hygiene directly impact performance?	Hospital leadership,infection control teams	Hospital administrations, research grants	Longitudinal studies, observational studies, intervention studies
**Leadership factors influencing commitment to hand hygiene improvement**	What leadership factors influence commitment to hand hygiene improvement?	Hospital leadership, research institutions	Government health departments, hospital administrations	Qualitative studies, leadership evaluations, surveys
**Relationship between national IPC programme and institutional safety climate**	How does a national IPC programme impact institutional safety climate?	Government health departments (responsible for IPC)	Government health departments, international health organizations	Policy analysis, case studies, surveys
**Relationship between patient participation and institutional safety climate**	How does patient participation influence institutional safety climate?	Patient groups, hospital management, infection control teams	Hospital administrations, NGOs	Patient surveys, focus groups, case studies
**Factors motivating decision-makers to involve patients in hand hygiene strategies**	What motivates decision-makers to involve patients in hand hygiene strategies?	Hospital management, patient representative groups	Research grants, hospital administrations	Qualitative studies, interviews, surveys
**Impact of patient participation on hand hygiene improvement**	How does patient participation affect hand hygiene improvement?	Patient representative groups, hospital leadership	Hospital administrations, NGOs	Intervention studies, case studies, surveys
**Role and impact of visitors and informal caregivers in hand hygiene improvement**	What role do visitors and informal caregivers play in hand hygiene improvement?	Patient representative groups, hospital leadership	Hospital administrations, research grants	Observational studies, surveys, intervention studies
**Perceptions of service users towards institutional safety climate**	How do service users perceive institutional safety climate?	Patient representative groups	Research grants, hospital administrations	Surveys, interviews, focus groups
**Relationship between national culture and patient participation in hand hygiene**	How does national culture affect patient participation in hand hygiene initiatives?	Behavioural scientists, policymakers, infection control teams	Research grants, international organizations	Cross-cultural studies, surveys, case studies

**Most effective methods of patient participation in hand hygiene practices**	What are the most effective methods for patient participation in hand hygiene practices?	Patient representative groups, behavioral/ implementation scientists	NGOs, hospital administrations	Intervention studies, surveys, qualitative studies
**Barriers and facilitators of patient participation in hand hygiene interventions**	What are the barriers and facilitators of patient participation in hand hygiene interventions?	Patient representative groups	Research grants, hospital administrations	Barrier and facilitator analysis, qualitative studies, surveys
**Influence of societal norms on institutional safety climate**	How do societal norms affect institutional safety climate?	Behavioural scientists, policymakers, infection control teams	Government health departments, hospital administrations	Sociocultural studies, qualitative research, surveys
